# ARDS and Massive Pulmonary Embolism: The Combined Use of Extracorporeal Membrane Oxygenation (ECMO) with Thrombolytics

**DOI:** 10.1155/2020/1032629

**Published:** 2020-04-28

**Authors:** Brett Dickens, Casey Bryant, John Gaillard, Nathaniel Westphal

**Affiliations:** Departments of Emergency Medicine and Anesthesiology, Wake Forest School of Medicine, Winston-Salem, NC, USA

## Abstract

Pancreatitis causes a systemic inflammatory response that can lead to acute respiratory distress syndrome (ARDS). We present a case of severe ARDS complicated by a pulmonary embolism (PE) in a 39-year-old female that developed rapidly progressive pancreatitis secondary to hypertriglyceridemia.

## 1. Introduction

Venovenous (VV) extracorporeal membrane oxygenation (ECMO) is a treatment option for management of severe ARDS. This report details a case of ARDS secondary to hypertriglyceridemia-induced pancreatitis complicated by obstructive shock secondary to a massive pulmonary embolism.

Our review of the literature regarding thrombolytic use while on ECMO revealed only three case studies and a single case series. Two of the three case reports were in neonates: myocardial infarction [1] and intracardiac thrombus [2]. The third case was from our facility and discussed catheter directed thrombolysis for a massive PE post-cardiac arrest while on venoarterial (VA) and later VV ECMO [3]. Finally, the case series consisted of 13 cases of massive PE at a single center where all patients were placed on VA ECMO. Eight patients received systemic thrombolytics, three received catheter-directed thrombolysis, and four underwent surgical embolectomy [4]. None of these cases discussed the use of thrombolytics on ECMO with acute pancreatitis.

## 2. Case Presentation

A 39-year-old female with history of Tourette syndrome, hypothyroidism, non-insulin-dependent diabetes mellitus (Hb A1c 6.3), obesity (BMI 38.7 kg/m^2^), and hyperlipidemia presented to a community emergency department for evaluation of abrupt onset abdominal pain, nausea, and vomiting. Her lipase level was 7,465 IU/L, and the computed tomography (CT) findings of her abdomen and pelvis were consistent with uncomplicated pancreatitis. Further workup revealed triglycerides of 7,121 mg/dL with no known history of hypertriglyceridemia in the past. She did not take any culprit medications for hypertriglyceridemia.

She was admitted to the community hospital for fluid resuscitation and was started on an insulin infusion. On hospital day 2, she became oliguric and developed dyspnea with bilateral infiltrates on chest x-ray concerning for ARDS. She was intubated later that day for hypoxic respiratory failure. Although an echocardiogram was not obtained at the outside hospital, her x-ray findings were not thought to be pulmonary edema due to heart failure by the clinicians caring for her at the time. On hospital day 3, the patient was transferred to our academic medical center for the management of ARDS requiring mechanical ventilation, pancreatitis, and worsening renal function. At the time of admission to our hospital, the triglyceride level had improved to 996 mg/dL, and the lipase level had improved to 923 IU/L on the insulin infusion. Although other modalities of treatment such as plasmapheresis have been shown to be efficacious in lowering triglycerides, this patient was continued on insulin infusion and IV fluid repletion given the significant decrease in triglyceride levels [5].

**Figure 1:**
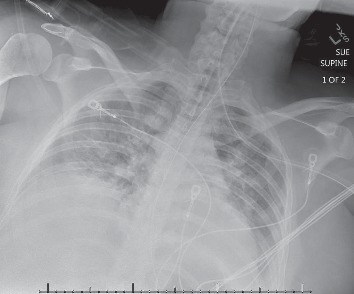
Chest x-ray.

**Figure 2:**
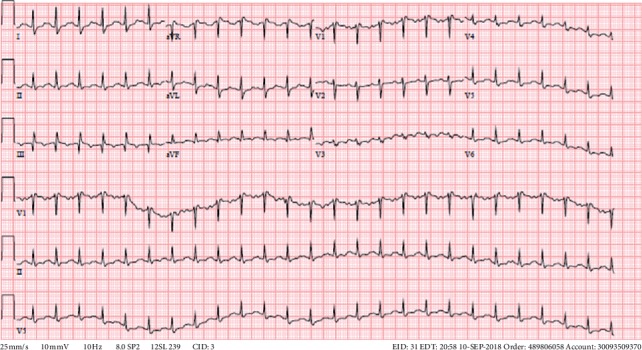
Electrocardiogram.

The patient arrived to our medical intensive care unit (MICU) overnight with severe ARDS (P/F of 90) secondary to pancreatitis (Figure 1). Vital signs included: blood pressure 122/63 mmHg, heart rate 150 beats per minute, respiratory rate 23 per minute, temperature 103.4°F (39.7°C), and pulse oximetry 95%. Ventilator settings were: assist control with set volume target 350 cc (∼6 cc/kg, IBW 59 kg), rate 26, FiO_2_ 100%, PEEP 16 cm H_2_O, PIP 38 cm H_2_O, Pplat 34. Arterial blood gas (ABG) on arrival was pH 7.16/pCO_2_ 46/pO_2_ 91. The base deficit was 12. Lactic acid was 3.32 mmol/L. The patient's ECG at the time of arrival revealed sinus tachycardia with HR in the 150 s, incomplete right bundle branch block (RBBB), and S1Q3T3 morphology (Figure 2). No prior ECG was available for comparison.

Over the next few hours, she became increasingly hypotensive with anuria. Her extremities were noted to be cool and dusky. Her peripheral pulses were only able to be obtained by the Doppler signal. Vasopressin and norepinephrine were used to maintain a mean arterial pressure of 65 mmHg. The patient was continued on an insulin drip, and a 10% dextrose/normal saline drip for treatment of the hypertriglyceridemia. She was also started on empiric broad-spectrum antibiotics because of concern for septic shock.

**Figure 3:**
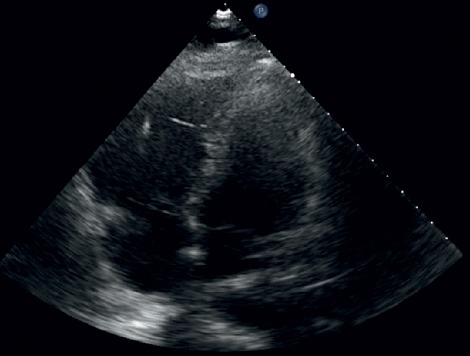
Echocardiogram.

Given the patient's hemodynamic instability on presentation, transthoracic echocardiogram was obtained that revealed a severely dilated right ventricle (RV) with moderately reduced function (Figure 3). In the parasternal short axis view, there was septal flattening in both systole and diastole consistent with elevated RV pressure and volume overload. The left ventricle (LV) was hyperdynamic with an ejection fraction of >70%. She was initiated on a heparin infusion at that time due to suspicion for pulmonary embolism.

Her hypoxia continued to worsen after arrival with oxygen saturations decreasing to 84–86%. The patient was sedated to a Richmond Agitation Sedation Score (RASS) of 5, paralyzed using cisatracurium and inhaled epoprostenol was initiated. Despite these treatments, her condition continued to worsen (norepinephrine up to 22 mcg/min and vasopressin 0.04 U/min) with ongoing hypoxia, so the decision was made to cannulate her for VV ECMO approximately 12 hours after admission to our hospital. Proning is performed at our center, but was not pursued due to the patient's worsening hemodynamics. Cannulation was performed with a 31 French Avalon bicaval cannula (Maquet, Rastatt, Germany) via the right internal jugular vein. The patient remained anuric for the next 24 hours with a rising BUN and Cr (initial BUN 12, Cr 1.44 increased to BUN 16, and Cr 2.16). Therefore, continuous renal replacement therapy (CRRT) was initiated for acute renal failure, which was believed to be due to acute tubular necrosis from shock and hypoxia.

At the time of cannulation, her condition was felt to be multifactorial with severe ARDS and distributive shock due to both sepsis and systemic inflammation from overwhelming pancreatitis. The right ventricular dilation and dysfunction was believed to be due to acidemia, high ventilator settings, and hypoxia. Obstructive shock due to acute PE was also high on the differential.

After cannulation for VV ECMO, the patient remained hypoxic and hemodynamically unstable. Bedside point of care ultrasound (POCUS) was performed that revealed continued RV dilation and decreased function that had not improved with the addition of epinephrine for inotropic support (5 mcg/min), correction of acidosis and hypoxia, and minimization of ventilator support (pressure control ventilation 20 with PEEP 10, rate 10, and FiO_2_ 40%). Bilateral lower extremity venous duplex ultrasounds revealed a thrombus in the right femoral vein. Due to high suspicion for PE contributing to the refractory shock and hypoxemic state, 50 mg of alteplase (tPA) (10 mg bolus, 40 mg infusion given over 2 hours) was administered systemically. Approximately 1 hour after completing the infusion of tPA, the patient's hemodynamics had improved to the point where vasopressors were weaned off and inotropic support had been titrated down to epinephrine 2 mcg/min. This concluded the events of hospital day 1 at our facility and day 3 of her total hospitalization.

The insulin infusion was discontinued on hospital day 5 once triglycerides dropped below 500 mg/dL and gemfibrozil was started. By day 5, the patient developed thrombocytopenia, which was concerning for heparin induced thrombocytopenia (HIT). Other considerations for the thrombocytopenia include CRRT, vancomycin-induced thrombocytopenia, and the use of epoprostenol. Argatroban was used for anticoagulation for 6 days until the HIT antibody screen and serotonin release assay were negative.

**Figure 4:**
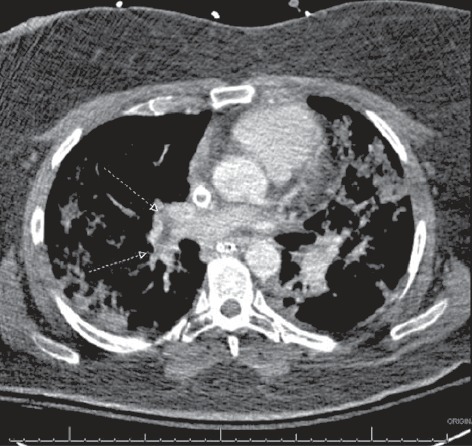
Computed tomography.

The patient continued to require VV ECMO for hypoxic respiratory failure despite improvement in the infiltrates on her chest x-rays. A CT angiogram of the chest was performed on hospital day 10 to evaluate the lung vasculature for suspicion of recurrent or persistent PE. Multiple pulmonary emboli were identified involving the distal right main pulmonary artery and multiple lobar pulmonary arteries (Figure 4). A CT of the abdomen/pelvis performed at the same time revealed extensive peripancreatic fluid collections and a thrombus in the portal vein extending into the splenic vein and superior mesenteric vein (SMV).

Due to the extensive venous thromboemboli and inability to wean from ECMO, interventional cardiology was consulted and performed catheter directed thrombolysis (CDT) via an EndoWave Infusion Catheter System (EKOS) on hospital day 11. The patient's hypoxia improved to the point where she was able to be decannulated from ECMO on hospital day 12. CRRT was continued until hospital day 13, at which time she was transitioned to intermittent hemodialysis. On hospital day 14, she was extubated. A repeat echocardiogram that day revealed an ejection fraction of 55–60% with normal size and function of the right ventricle. On hospital day 18, she transferred out of the intensive care unit.

Due to extensive diffuse venous thromboemboli, hypercoagulability studies were sent including antithrombin III, protein C and S, antinuclear antibody screen, cardiolipin IgA/IgM/IgG, plasminogen activity, and thrombophilia genotype panel (including factor V Leiden). The patient was heterozygous for factor II 20210 mutation. Hematology was consulted and reported this mutation alone does not typically require anticoagulation, because the mutation does not often lead to VTE. In this case, the mutation was thought to be contributory to the patient's significant thromboemboli likely induced by the severe systemic inflammatory response caused by pancreatitis. Hematology recommended indefinite anticoagulation.

The patient's course was also unfortunately complicated by limb ischemia, believed to be due to microthrombi from her hypercoagulable state and her concomitant vasopressor requirement. Ultimately, she required bilateral below knee amputations for dry gangrene. Expectant management was pursued for the gangrenous distal fingertip injuries. She completed a stay in acute rehabilitation, and approximately 3 months after discharge from rehab the patient returned for carpal tunnel release surgery, eschar removal of the left index finger, and amputation through the right ring finger at the DIP joint. She was also scheduled to follow-up regarding bilateral lower extremity prosthesis placement and fitting. Approximately 7 months after the date of admission to our hospital, the patient reached out to team members through social media and indicated that she was doing well and progressing in her rehabilitation. She has since been lost to any further follow-up.

## 3. Discussion

Our team acknowledges that VA ECMO is the extracorporeal treatment of choice when pulmonary embolism is the cause of cardiogenic shock [6]. Our patient was placed on VV ECMO as the initial hypothesis was that her cor pulmonale was due to her critical illness and high ventilator settings. When her RV function did not improve after optimizing these conditions, the search for alternative explanations of her illness continued. She was ultimately given thrombolytics due to a high clinical suspicion for PE. Transitioning to VAV support was also discussed, but this was decided against due to the peripheral limb ischemia that she was already demonstrating. This case highlights the complex and high stakes nature of caring for critically ill patients as well as the importance of assessing treatments for response and continuing the pursuit of alternative explanations when patients do not respond as expected to interventions.

While acute pancreatitis is a common disease, there are few documented cases of PE in this patient population. Vascular complications associated with pancreatitis are thought to be related to the release of proteolytic enzymes that cause vessel erosion allowing for pseudocyst formation and endothelial disruption resulting in local vasculitis, ultimately leading to an increased risk of thrombosis [7]. There is an increased risk of hemorrhage associated with these vascular complications, particularly when there is pseudocyst formation. Life threatening bleeding can occur.

There are few case reports of patients receiving thrombolysis during an episode of acute pancreatitis. Review of the literature revealed once such case which involved a 54-year-old man initially misdiagnosed with STEMI who received thrombolytics with no life-threatening complications [8]. The second case discussed a 47-year-old man with acute alcoholic pancreatitis who received thrombolytics for STEMI and subsequently died of retroperitoneal hemorrhage from pseudocyst rupture [9]. Additionally, a single case was published that discussed the successful use of thrombolysis in a patient with both a pulmonary embolism and acute pancreatitis [7].

The standard dose of tPA for massive PE is 100 mg infused over 2 hours. However, literature review did not reveal a standard dose for a patient on VV ECMO, and the diagnosis in our patient had not been confirmed. Additionally, there was concern for possible hemorrhagic conversion of pancreatitis or other complications given the patient's complexity. In this case, the reduced dose of 50 mg (10 mg bolus, 40 mg infusion over 2 hours) proved efficacious and improved hemodynamics immediately with no adverse effects. However, the patient did later require CDT via EKOS catheter for further treatment of persistent pulmonary emboli that were prohibiting her from weaning off ECMO support. The increased volume of distribution contributed by the ECMO circuit may have contributed to the decreased overall efficacy of the initial dose of thrombolytics.

Standard dosing for tPA was extrapolated for use in PE. Importantly, when tPA is given for ailments such as stroke, only a fraction of the total blood volume may enter the circulation of the affected organ (15% of the total circulation in the case of the brain). This is much different than the 100% of blood volume that cycles through the pulmonary vasculature. Recent studies have supported a decreased dose with improved safety profile, though the topic of treating submassive PE with thrombolytics remains controversial. The Moderate Pulmonary Embolism Treated with Thrombolysis (MOPETT) trial illustrated a trend towards safe and effective tPA administration at lower than normal dosing for submassive PE [10]. The Pulmonary Embolism Thrombolysis (PEITHO) trial demonstrated a trend towards reduced cardiac complications (death or hemodynamic instability) at 7 days after administration of full dose tenecteplase for submassive PE. In that study of over 1,000 patients, death and cardiovascular collapse were decreased by nearly 56% by using tenecteplase plus heparin compared to heparin alone; however, major bleeding and hemorrhagic stroke increased by as much as 10-fold [11]. In an attempt to bypass such bleeding complications, catheter-directed therapy was proposed as an alternative. The SEATTLE II study, published in 2015, showed that catheter-directed therapy of thrombolytics for submassive PE could effectively reduce RV dilation, pulmonary hypertension, and anatomic thrombus burden while also possibly minimizing intracranial hemorrhage [12]. While catheter-directed thrombolysis has proven effective in submassive PE, it has never been compared in a head to head trial against systemic thrombolytics so the topic continues to be debated. In summary, for submassive PE, the indication, appropriate dose, and route of thrombolytic treatment all remain controversial.

## 4. Conclusion

This case highlights the complexity in managing critically ill patients with multiple acute comorbidities including hypercoagulability, ARDS, obstructive shock, and acute pancreatitis each complicating the available treatment options and goals in therapy. Additionally, it highlights the importance of continued studies looking at thrombolytic administration in both ECMO patients as well as acute pancreatitis.
